# Tackling the Threat of Cancer Due to Pathobionts Producing Colibactin: Is Mesalamine the Magic Bullet?

**DOI:** 10.3390/toxins13120897

**Published:** 2021-12-14

**Authors:** Min Tang-Fichaux, Priscilla Branchu, Jean-Philippe Nougayrède, Eric Oswald

**Affiliations:** 1 IRSD, Université de Toulouse, INSERM, INRAE, ENVT, UPS, 31024 Toulouse, France; min.tang@inserm.fr (M.T.-F.); priscilla.branchu@inserm.fr (P.B.); jean-philippe.nougayrede@inserm.fr (J.-P.N.); 2 Service de Bactériology-Hygiène, Hôpital Purpan, CHU de Toulouse, 31059 Toulouse, France

**Keywords:** colibactin, polyphosphate kinase, mesalamine, inflammatory bowel disease, colorectal cancer

## Abstract

Colibactin is a genotoxin produced primarily by *Escherichia coli* harboring the genomic *pks* island (*pks*^+^ *E. coli*). *Pks*^+^ *E. coli* cause host cell DNA damage, leading to chromosomal instability and gene mutations. The signature of colibactin-induced mutations has been described and found in human colorectal cancer (CRC) genomes. An inflamed intestinal environment drives the expansion of *pks*^+^ *E. coli* and promotes tumorigenesis. Mesalamine (i.e., 5-aminosalycilic acid), an effective anti-inflammatory drug, is an inhibitor of the bacterial polyphosphate kinase (PPK). This drug not only inhibits the production of intestinal inflammatory mediators and the proliferation of CRC cells, but also limits the abundance of *E. coli* in the gut microbiota and diminishes the production of colibactin. Here, we describe the link between intestinal inflammation and colorectal cancer induced by *pks*^+^ *E. coli*. We discuss the potential mechanisms of the pleiotropic role of mesalamine in treating both inflammatory bowel diseases and reducing the risk of CRC due to *pks*^+^ *E. coli*.

## 1. Introduction

*Escherichia coli* is a ubiquitous intestinal commensal and one of the most frequently isolated organisms from clinical specimens. Of all *E. coli* strains, phylogenetic group B2 strains are increasingly found in the feces of healthy humans in high-income countries [1,2]. *E. coli* group B2 includes commensal strains and pathogenic strains that are responsible for extra-intestinal diseases, including urinary tract infections, sepsis, pneumonia, and neonatal meningitis. Among the strains of the B2 phylogroup, there are a large number of strains that possess in their genome the *pks* island that encodes the genotoxin colibactin [3,4]. These *pks*^+^ strains of *E. coli* are frequently found in asymptomatic carriage in the digestive tract from the first days after birth [5,6]. As we will show in this review, the intestinal genotoxicity of these bacteria greatly depends on the intestinal environment such as the presence of certain food contaminants [7], and especially on the inflammatory response, which suggests that these so-called commensal strains of *E. coli* are in fact true pathobionts.

The *pks* island encoding enzymes responsible for colibactin biosynthesis is mainly present in this group. The sequence of the *pks* island is highly conserved and *pks*-carrying (*pks*^+^) strains are found overwhelmingly capable to produce colibactin [3]. The biosynthesis of colibactin has been summarized in two nice reviews [8,9]. Colibactin is a secondary metabolite belonging to the chemical family of hybrid polyketide (PK)–non-ribosomal peptides (NRP). Colibactin directly damages host DNA both in vitro [10,11,12] and in vivo [5,13,14,15,16,17,18,19]. This genotoxin is linked to the virulence of pathogenic *E. coli* [15,17,20], intestinal microbial diversity [21] and intestinal homeostasis [5,22]. Importantly, accumulating evidence suggests that the pathobiont *pks*^+^ *E. coli* is associated with colorectal cancer (CRC) [10,16,18,23,24,25,26,27]. CRC is the third most commonly diagnosed cancer and the fourth leading cause of cancer-related deaths worldwide, and its burden is expected to increase by 60% to more than 2.2 million new cases and 1.1 million annual deaths by 2030 [28]. CRC has long been known to be associated with chronic intestinal inflammation, such as inflammatory bowel disease (IBD) [29]. IBD is associated with gut microbiota dysbiosis, characterized by the increased abundance of facultative anaerobic Enterobacteriaceae that include *pks*^+^ *E. coli* [30].

Mesalamine (i.e., 5-aminosalycilic acid), an anti-inflammatory drug, has been in the first-line therapy for IBD for decades [31]. A long-term use of mesalamine has chemopreventive properties against CRC [32,33,34]. The exact mechanism of action of mesalamine is unknown. However, it has been shown that, on one hand, mesalamine inhibits host inflammatory cascades and inhibits the proliferation of CRC cells [35,36,37,38]. On another hand, mesalamine alters bacterial microbiota composition, which is characterized by the reduction of Enterobacteriaceae (e.g., *E. coli*) in inflamed mucosa [39,40]. Interestingly, mesalamine is an inhibitor of the bacterial polyphosphate kinase (PPK) [41]. PPK is essential for the synthesis of long-chain polyphosphate (polyP) [42]. The treatment with mesalamine decreases polyphosphate (polyP) levels in gut microbiota, which may sensitize bacteria to oxidative stress [43] and diminish bacterial persistence within the inflamed mucosa. We have recently shown that mesalamine directly reduces colibactin production by *pks*^+^ *E. coli* in a PPK-dependent/ independent manner [44]. In this review, we describe the association of *pks*^+^ *E. coli* and intestinal inflammation in the development of CRC, and we discuss the pleiotropic role of mesalamine in the prevention of CRC due to bacteria producing colibactin.

## 2. *Pks*^+^ *E. coli* Induce DNA Damage and Have a Mutational Impact in Colorectal Cancer

**Figure 1:**
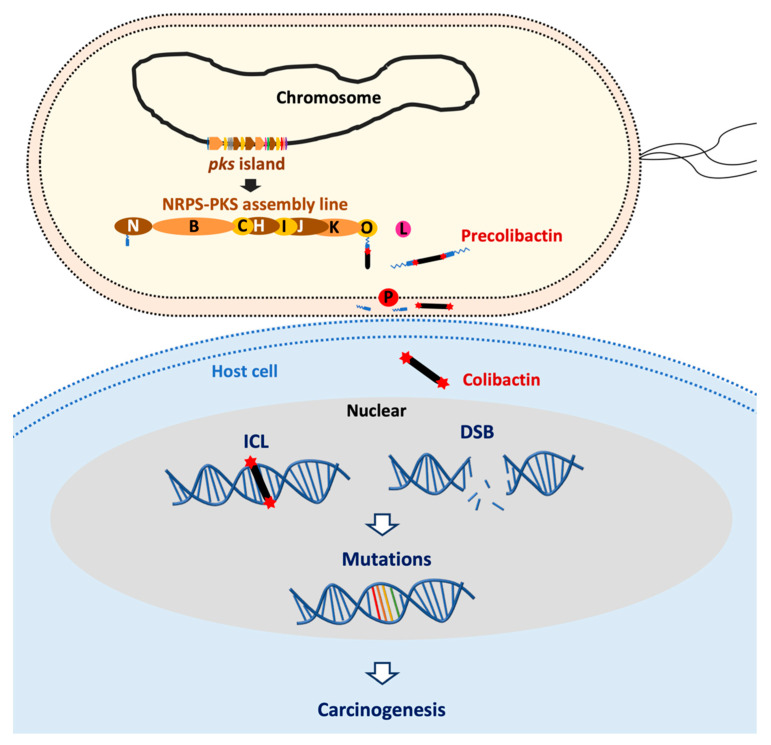
*Pks*^+^ *E. coli* induce DNA damage and have a mutational impact leading to cancer formation. Colibactin is synthesized by an assembly line composed of non-ribosomal peptide synthetase (NRPS) and polyketide synthases (PKS) which are encoded by the *pks* island in *E. coli*. Colibactin produced by *pks*^+^ *E. coli* causes DNA inter-strand cross-links (ICLs) in a cell-to-cell contact dependent manner. ICLs *in cellulo* are converted into DNA double-strand breaks (DSBs) during the DNA-damage response or by depurination. DNA damages potentially can result in gene mutations which may trigger carcinogenesis.

Unlike other genotoxins such as CDT, colibactin is not a proteinaceous “exotoxin” [45]. Colibactin is a hybrid PK-NRP compound which has a nearly symmetrical structure that contains two electrophilic cyclopropane warheads [46]. The warheads have a high binding-affinity for adenine residues within AAWWTT nucleotide motifs [10,27]. The warheads alkylate DNA on two adenine residues of opposite strands of DNA, resulting in DNA inter-strand cross-links (ICLs) [11,14,46,47]. How exactly the host cells repair colibactin-induced DNA damage and remove the ICLs remains to be studied, but ICLs are converted into DNA double-strand breaks (DSBs) during the DNA-damage response (DDR), or evolve directly to DSBs by depurination [48] (Figure 1).

DNA damage caused by colibactin has been observed *in cellulo* [10,11,12] and in mouse models of digestive or urinary tract colonization [5,13,14,15,16,17,18,19]. The consequences for cells infected by *pks*^+^ *E. coli* depend on the infection dose: A high infectious dose induces massive DNA damage, resulting in mitotic catastrophe and apoptosis [11,12,19], whereas a milder infectious dose induces persistent DNA damage and senescence [16,49]. With a low infectious dose, cells repair most of the damage and resume their cell cycle, but with the establishment of a chromosomal instability phenotype and a significant increase in gene mutation frequency [19] (Figure 1).

Recent reports have revealed the distinct mutational signature caused by *pks*^+^ *E. coli* (also called the colibactin footprint), and the same mutational signature has been detected in human cancer genomes, predominantly in CRC [10,27,50,51]. Among the common CRC driver mutations, 2.4–9% match the colibactin footprint, and the adenomatous polyposis coli (APC) gene *APC* (a tumor-suppressor gene) contains the highest number of mutation matches [10,27]. Nevertheless, we should recognize that the colibactin footprint in CRC genomes and corresponding driver genes may represent only the tip of the iceberg of the impact of *pks*^+^ *E. coli* [52]. A recent study has demonstrated that infection with *pks*^+^ *E. coli* fails to induce a typical colibactin mutational signature, but the genotoxic effect is sufficient to rapidly cause chromosomal aberrations in primary colon cells and to induce multiple features of transformation reminiscent of CRC [53]. This may be due to the failure of resolving ICL via repair machinery prior to mitosis. Consistent with this notion, we have indeed observed chromosomal aberrations after a short-time infection in vitro [19].

Collectively, these findings indicate the mutagenic potential of *pks*^+^ *E. coli* and its causative role in CRC. Nevertheless, we should also recognize that the presence of *pks*^+^ *E. coli* may be not enough for the development of CRC but exhibits its carcinogenic role under certain conditions such as inflammation.

## 3. Intestinal Inflammation, *pks*^+^ *E. coli* and Colorectal Cancer

### 3.1. Intestinal Inflammation Contributes to Colorectal Cancer

**Figure 2:**
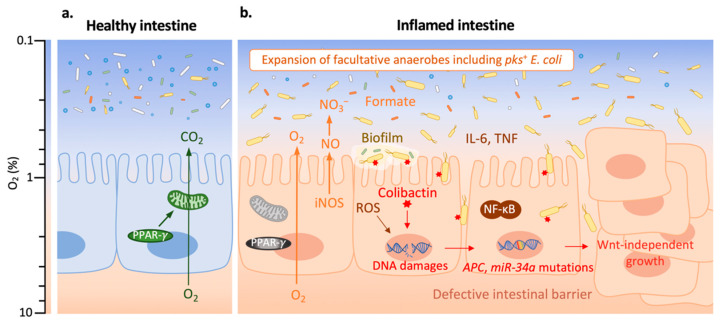
*Pks*^+^ *E. coli* promote tumorigenesis in an inflamed environment. Adapted from reference [64].The color scale on the left indicates oxygen (O_2_) levels. (**a**) The healthy intestine harbors a diverse and dynamic microbial community (microbiota) which is dominated by obligate anaerobes (in white, green or blue); the expression of peroxisome proliferator-activated receptor gamma (PPAR-γ) in intestinal epithelial cells (IECs) activates mitochondrial bioenergetics that consume O_2_, and thereby maintains epithelial hypoxia (O_2_ level < 1%). (**b**) In the inflamed intestine, nuclear factor kappa B (NF-κB) plays an important role for the transition from inflammation to malignancy. NF-κB induces interleukin 6 (IL-6) which promotes IEC proliferation, the inhibition of apoptosis, and other pro-tumorigenic pathway; the activation of tumor necrosis factor (TNF) enhances the activation of NF-κB, resulting in de-differentiation of IEC and facilitation of tumor initiation. NF-κB induces the generation of reactive oxygen species (ROS) which increase epithelial oxygenation. A lower PPAR-γ expression also leads to a higher epithelial oxygenation. The increased O_2_ level benefits the expansion of facultative anaerobic Enterobacteriaceae, including *pks*^+^ *E. coli* (in yellow; other Enterobacteriaceae are in orange). Inducible nitric oxide synthase (iNOS) is expressed at a high level during intestinal inflammation, resulting in an elevated production of nitric oxide (NO) which generates nitrate (NO_3_^−^); NO_3_^−^ boosts the growth of *pks*^+^ *E. coli*. The increased concentration of microbiota-derived formate enhances *E. coli* fitness in the inflamed intestine. Biofilm formation and intestinal barrier dysfunction are often found in colitis, which promote *pks*^+^ *E. coli* contact with IEC and invasion. Colibactin produced by *pks*^+^ *E. coli* causes DNA damages which lead to the mutations in genes, such as *APC* and *miR-34a*. This results in colorectal cancer (CRC) cells which are characterized by Wnt-independent growth, enhanced proliferation and impaired differentiation.

Intestinal inflammation is an important contributor to CRC formation [29,54]. Patients with IBD, such as Crohn’s disease (CD) or ulcerative colitis (UC), have a higher risk of developing CRC. These incidence rates of CRC in IBD patients correspond to cumulative probabilities of 2% by 10 years, 8% by 20 years, and 18% by 30 years [55]. The underlying mechanisms of the transition from inflammation to malignancy have not yet been fully understood. Nuclear factor kappa B (NF-κB), a key transcription factor that regulates immunological and inflammatory responses, has been identified as one of the main participants in this transition [56] (Figure 2). One of the main pro-inflammatory cytokines induced by NF-κB is interleukin 6 (IL-6), which activates the transcription factor STAT3 in intestinal epithelial cells (IECs) and then promotes tumorigenesis by facilitating their proliferation, the inhibition of apoptosis, and/ or other pro-tumorigenic pathways [57]. This IL-6-STAT3 signaling can also repress the tumor-suppressor miR-34a [58]. Another pro-inflammatory cytokine involved in NF-κB activation is tumor necrosis factor (TNF). Constitutive activation of Wnt induces the activation of TNF that enhances activation of NF-κB, resulting in the de-differentiation of IECs and facilitation of tumor initiation [59]. Moreover, mutation of the gene encoding p53 (*Tp53*), an early event in inflammation-associated CRC [60], causes NF-κB activation in IECs and surrounding stromal cells and induces epithelial–mesenchymal transition [61,62]. Constitutive activation of NF-κB induces DNA damage via the generation of reactive oxygen species (ROS) [63], which provides an additional link between chronic inflammation and tumorigenesis.

### 3.2. Intestinal Inflammation Promotes the Carcinogenic Activity of pks^+^ E. coli

While intestinal inflammation *per se* contributes to CRC, intestinal inflammation is associated with the perturbation of gut microbiota composition and activities, which can further promote inflammation and cancer development [65]. Here, we focus on discussing the pro-tumorigenic interplay between inflammation and *pks*^+^ *E. coli*. In mouse models of inflammation-associated CRC, *pks*^+^ *E. coli* have been shown to enhance tumorigenesis [16,18,26,66]. Inflammation is essential for *pks*^+^ *E. coli*-induced CRC, as *pks*^+^ *E. coli* failed to induce CRC in inflammation-resistant mice [67]. Inflammation could favor *pks*^+^ *E. coli* carcinogenic activity (i.e., colibactin-induced DNA damage in IECs) by facilitating *pks*^+^ *E. coli* expansion, enhancing their attachment to the mucosa, or/and increasing the expression of *pks* genes (Figure 2).

During intestinal inflammation, epithelial-derived ROS not only induce DNA damage as mentioned above, but also increase mucosal oxygenation, which favors the aerobic expansion of *E. coli* [68]. In addition to ROS, less oxygen consumption by IECs during inflammation also elevates mucosal oxygenation [64]. Peroxisome proliferator-activated receptor gamma (PPAR-γ) has a lower expression in IECs of IBD patients compared with healthy subjects [69,70]. PPAR-γ plays an important role in activating mitochondrial bioenergetics that causes high oxygen consumption, and thereby maintains epithelial hypoxia [71]. Therefore, a lower expression of PPAR-γ in IECs of IBD patients leads to a higher epithelial oxygenation that benefits the expansion of facultative anaerobes Enterobacteriaceae, including *pks*^+^ *E. coli*. Importantly, the aerobic expansion of *pks*^+^ *E. coli* is required for the cancer-inducing activity of this pathobiont in a mouse model of inflammation-associated CRC [24]. Moreover, the high-level production of inflammation-derived [72] nitrate and microbiota-derived formate [73] in the inflamed intestine also benefits the outgrowth of Enterobacteriaceae. It has been shown that the administration of tungstate blunts the expansion of Enterobacteriaceae to ameliorate colitis [74]. Further, it has been identified that tungstate treatment decreases the intestinal burden of *pks*^+^ *E. coli*, reduces DNA damage, and thereby lowers tumor incidences [75].

In addition to the *E. coli* expansion, the cancer-inducing activity of *pks*^+^ *E. coli* may also require mucosal invasion to have a direct contact with IECs, according to in vitro evidence that the genotoxicity of *pks*^+^ *E. coli* is cell-to-cell contact-dependent [12]. Interestingly, intestinal inflammation might favor the contact of *pks*^+^ *E. coli* to IECs by enhancing mucosal invasion of this pathobiont. Bacterial biofilms which can promote bacterial mucosal invasion are often found in patients with IBD or CRC [76,77]. *Pks*^+^ *E. coli* have shown a higher prevalence (68%) in mucosal biofilms from CRC patients than from healthy subjects [26]. Noticeably, *pks*^+^ *E. coli* are indeed more frequently found in stool or intestinal biopsy samples from IBD/CRC patients than from healthy subjects [18,26,78,79]. IBD patients as well as mouse models mimicking the human disease often show defects in intestinal barrier function, resulting in an increase of mucus penetration by bacteria [80]. Mucosal invasion may not only allow the translocation of colibactin from bacteria to IECs, but also help the invasion of some *pks*^+^ *E. coli* strains into host cells, which may exacerbate the inflammatory response, enhance bacterial persistence, and exacerbate genotoxicity. Raisch et al. has demonstrated that *pks*^+^ *E. coli* are able to resist killing by human THP-1 macrophages, to replicate intracellularly, and to persist inside host cells until at least 72 h after infection, which significantly increases the production of the protumoral factor cyclooxygenase-2 (COX-2) [81]. Still, whether intracellular *pks*^+^ *E. coli* cause DNA damage and induce a bystander effect requires future studies to identify.

In some cases, neither fecal nor tissue-associated *pks*^+^ *E. coli* abundance was significantly changed with inflammation, but the overall expression of the *pks* island was significantly higher when inflammation was higher [82]. This is consistent with another report which shows that the expression of *pks* genes (*clbG*, *clbH*, *clbL*, *clbM* and *clbN*) is upregulated in an inflamed environment [67]. The increased expression of *pks* genes could account for another role of inflammation in *pks*^+^ *E. coli* carcinogenic activity.

Altogether, these findings suggest that inflammation and *pks*^+^ *E. coli* synergistically promote CRC formation. Tumorigenesis is driven, at least partially, by selective enrichment or/and invasion of *pks*^+^ *E. coli* in the inflamed environment. Intestinal inflammation may also drive tumorigenesis by upregulating the expression of *pks* genes, presumably leading to more colibactin production, and thereby more DNA damage in the colon epithelium.

## 4. The Roles of Mesalamine in the Treatment of Inflammation and the Prevention of Cancer Promoted by *pks*^+^ *E. coli*

**Figure 3:**
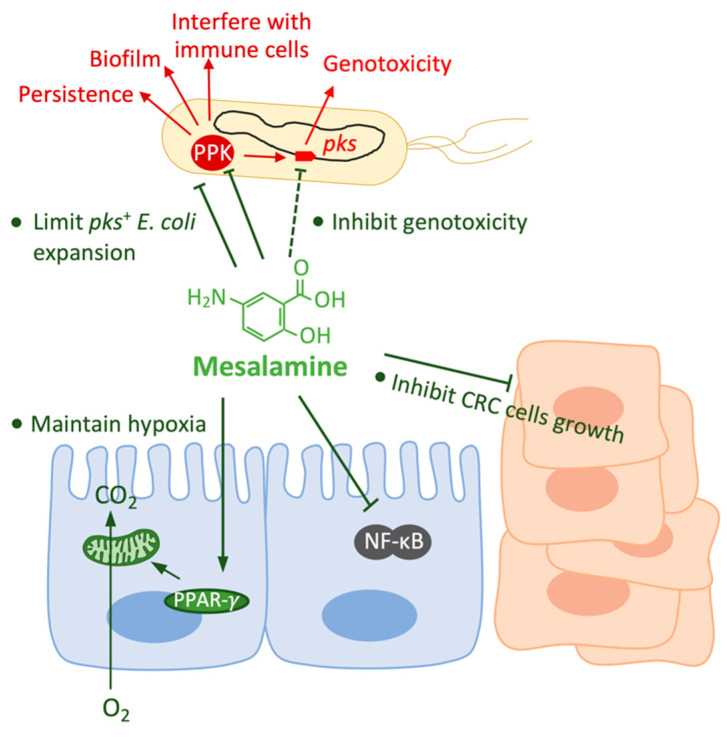
The pleiotropic effects of mesalamine on the host and on *pks*^+^ *E. coli*. Mesalamine has effects on both host cells and *pks*^+^ *E. coli*. Mesalamine inhibits the growth and enhances the death of CRC cells; mesalamine interferes with the activation of nuclear factor kappa B (NF-κB) which plays an important role for the transition from inflammation to malignancy; and, mesalamine-driven PPAR-γ activation restores mitochondrial bioenergetics that consume O_2_, and thereby restore epithelial hypoxia. Mesalamine limits the expansion of *pks*^+^ *E. coli* by reducing epithelial oxygenation or directly inhibiting the aerobic growth. Mesalamine is an inhibitor of polyphosphate kinase (PPK), by which mesalamine may inhibit various of bacterial abilities, such as persistence, biofilm formation and interference with immune cells; and mesalamine inhibits colibactin production in a PPK-dependent and independent way.

The anti-inflammatory drug mesalamine, a structural analogue of aspirin (Figure 3), is the first-line treatment of mild to moderate flares of UC and, especially, for maintenance of remission [83]. For off-label use, clinicians may use mesalamine in CD after surgical resection of the affected bowel. Moreover, long-term mesalamine therapy has been suggested for reducing the risk of IBD-related CRC [32,33,34].

### 4.1. Effects of Mesalamine on the Host

Mesalamine has direct effects on host cells (Figure 3). Mesalamine acts predominantly at the site of inflammation and its efficacy is dependent on achieving high intraluminal concentrations [84]. It has been shown that mesalamine downregulates the COX-2/PGE2 axis in inflammatory cells [85,86] and CRC cells [38]. Mesalamine arrests the growth and enhances the death of CRC cells in a COX-2-dependent/independent manner [38]. Moreover, it has been shown that mesalamine interferes with NF-κB activation in mouse colonic epithelial cells and CRC cells [87,88]. Furthermore, mesalamine has been shown to inhibit Wnt/β-catenin pathway both in CRC cells [89,90] and in vivo [91,92]. In addition to the inhibitory effects of mesalamine described above, mesalamine has been well known as a PPAR-γ agonist [37,70]; it has been shown to restore PPAR-γ in the colonic mucosa [93]. The mesalamine-driven PPAR-γ activation induces the apoptosis and growth inhibition of CRC cells [94].

### 4.2. Effects of Mesalamine on pks^+^ E. coli

Mesalamine treatment has effects not only on host cells, but also on bacteria. Here we focus especially on *pks*^+^ *E. coli* (Figure 3). So far, the most well-described direct effect of mesalamine on bacteria is the inhibition of bacterial PPK [41]. Our recent study has demonstrated that the inactivation of PPK by mutagenesis reduces the promoter activity of *clbB* (one of the essential enzymes for colibactin biosynthesis), and decreases the production level of colibactin [44]. We have therefore used mesalamine to treat *pks*^+^ *E. coli* and found the same effects than PPK inactivation. However, the production of colibactin in the mutant lacking PPK is further reduced by mesalamine, indicating that mesalamine is capable of inhibiting colibactin production independently from its inhibitory effect on PPK. Our results are consistent with an earlier report which shows that mesalamine inhibits the transcription of *pks* genes, and reduces DSBs caused by *pks*^+^ *E. coli* [95]. Thus, these results suggest that the chemopreventive effect of mesalamine, at least partially, relies on its inhibitory effect on colibactin production by *pks*^+^ *E. coli*, and thereby reduces DNA damage in IECs.

PPK, besides colibactin production, is also linked to many other important bacterial activities [42], e.g., resistance to oxidative stress [43] as well as biofilm formation and resistance to antibiotics [96]. Recent studies have even shown that polyP disturbs multiple macrophage functions for evading host immunity [97]. Mesalamine treatment sensitizes *E. coli* to inflammatory oxidant, reduces biofilm formation, and decreases bacterial persistence [41]. Mesalamine has also been shown to inhibit directly the growth of *pks*^+^ *E. coli* in aerobic conditions [39,95]. Moreover, instead of a direct inhibitory effect on bacteria, mesalamine limits the aerobic expansion of *pks*^+^ *E. coli* through an indirect way, whereby mesalamine-driven activation of PPAR-γ restores epithelium hypoxia [39]. Therefore, mesalamine could reduce *pks*^+^ *E. coli* carcinogenic activity not only by inhibiting the production of colibactin, but also by limiting *pks*^+^ *E. coli* colonization and invasion in an inflamed environment, presumably reducing the contact of *pks*^+^ *E. coli* to IECs.

Collectively, these data suggest that the chemopreventive effect of mesalamine not only depends on its ability to ameliorate the severity of inflammation, but also to inhibit the carcinogenic activity of *pks*^+^ *E. coli*.

## 5. Conclusions

Accumulating evidence indicates that *pks*^+^ *E. coli* is associated with CRC. Notably, an inflammation environment is required for the cancer-inducing activity of *pks*^+^ *E. coli* [16,18,26,66]. Intestinal inflammation favors the expansion and mucosal invasion of *pks*^+^ *E. coli* by increasing epithelial oxygenation [24], promoting bacterial biofilm formation [76], or decreasing mucosal barrier integrity [80]. Moreover, inflammation enhances the expression of *pks* genes [67,82]. In turn, *pks*^+^ *E. coli* strains exacerbate inflammation and induce DNA damage, and thereby promote tumorigenesis. Therefore, an efficient therapy to treat IBD and prevent CRC should include both anti-inflammation and anti-*pks*^+^ *E. coli* activities. The anti-inflammatory drug mesalamine has been used as a gold standard to treat UC and has been shown to prevent CRC. Additionally, non-steroidal anti-inflammatory drugs (NSAIDs), especially COX-2 inhibitors such as aspirin, are thought to be potentially valuable anti-tumor agents [98]. However, due to the potential harm to cardiovascular, renal, hepatic, and gastrointestinal tissues, the usage of NSAIDs should be cautious [99]. Given the involvement of gut microbiota composition and activities in CRC development, the impact of NSAIDs on the microbiota should be evaluated.

**Figure 4:**
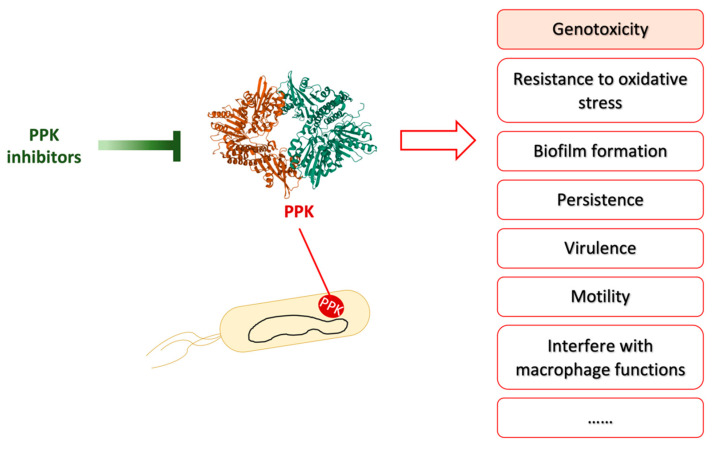
PPK inhibitors inhibit PPK activity to repress various bacterial activities. Adapted from reference [101]. PPK is involved in many bacterial abilities, such as genotoxicity, resistance to oxidative stress, biofilm formation, biofilm formation, persistence, virulence, motility, and interfere with macrophage functions etc. PPK inhibitors have been shown to inhibit some of these bacterial abilities in vitro or/and in vivo. The crystal structure of polyphosphate kinase (PPK) was downloaded from Protein Data Bank under the accession codes 1XDO [102].

Mesalamine directly interferes with bacterial functions via inhibiting PPK enzyme activity and other unknown mechanisms. Mesalamine treatment not only diminishes the abilities of *pks*^+^ *E. coli* linked to persistence or invasion (i.e., resistance to oxidative stress, biofilm formation, and resistance to antibiotics) [41], but also reduces the genotoxicity of *pks*^+^ *E. coli* [44]. PPK, as the essential enzyme for long-chain polyP synthesis in bacteria, plays many important roles in various bacterial functions which have been summarized in a very nice, recent review [42] (Figure 4). Therefore, mesalamine may affect other PPK-associated abilities. Besides mesalamine, there are many other PPK inhibitors that have been identified [42,100]. These inhibitors may also be able to reduce the genotoxicity of *pks*^+^ *E. coli*, and might have the potential to reduce the risk of CRC development due to *pks*^+^ *E. coli*. In addition, if the PPK inhibitor also has an anti-inflammatory effect, it might be the next “mesalamine”, which could benefit patients who are intolerant to mesalamine.

## Data Availability

No new data were created or analyzed in this study. Data sharing is not applicable to this article.
